# The Roles of Exosomes in Visual and Auditory Systems

**DOI:** 10.3389/fbioe.2020.00525

**Published:** 2020-06-03

**Authors:** Pei Jiang, Shasha Zhang, Cheng Cheng, Song Gao, Mingliang Tang, Ling Lu, Guang Yang, Renjie Chai

**Affiliations:** ^1^MOE Key Laboratory for Developmental Genes and Human Disease, School of Life Science and Technology, Jiangsu Province High-Tech Key Laboratory for Bio-Medical Research, Southeast University, Nanjing, China; ^2^Department of Otolaryngology Head and Neck Surgery, Nanjing Drum Tower Hospital Clinical College of Nanjing Medical University, Jiangsu Provincial Key Medical Discipline (Laboratory), Nanjing, China; ^3^Research Institute of Otolaryngology, Nanjing, China; ^4^Department of Otolaryngology, Affiliated People’s Hospital of Jiangsu University, Zhenjiang, China; ^5^Co-Innovation Center of Neuroregeneration, Nantong University, Nantong, China; ^6^Institute for Cardiovascular Science, Department of Cardiovascular Surgery of the First Affiliated Hospital, Medical College, Soochow University, Suzhou, China; ^7^Department of Otorhinolaryngology, Affiliated Sixth People’s Hospital of Shanghai Jiao Tong University, Shanghai, China; ^8^Institute for Stem Cell and Regeneration, Chinese Academy of Sciences, Beijing, China

**Keywords:** exosome, biogenesis, composition, isolation, eye, hair cell

## Abstract

Exosomes are nanoscale membrane-enclosed vesicles 30–150 nm in diameter that are originated from a number of type cells by the endocytic pathway and consist of proteins, lipids, RNA, and DNA. Although, exosomes were initially considered to be cellular waste, they have gradually been recognized to join in cell-cell communication and cell signal transmission. In addition, exosomal contents can be applied as biomarkers for clinical judgment and exosomes can as potential carriers in a novel drug delivery system. Unfortunately, purification methods of exosomes remain an obstacle. We described some common purification methods and highlight *Morpho Menelaus (M. Menelaus)* butterfly wings can be developed as efficient methods for exosome isolation. Furthermore, the current research on exosomes mainly focused on their roles in cancer, while related studies on exosomes in the visual and auditory systems are limited. Here we reviewed the biogenesis and contents of exosomes. And more importantly, we summarized the roles of exosomes and provided prospective for exosome research in the visual and auditory systems.

## Introduction

Extracellular vesicles (EVs) belong to a class of phospholipid bilayer membrane enclosed vesicles, and they are classified according to their biological origin and size (Shao et al., 2018; van Niel et al., 2018). Over the past decade, EVs have been found adjust and boost intercellular communication (El Andaloussi et al., 2013b), including signal transduction between cells, and thus invovled in cell proliferation, cell migration, immune regulation, etc (Meldolesi, 2018; Seo et al., 2018; Zhang S. et al., 2018). EVs are divided into three main types by their biological processes: exosomes, microvesicles and apoptotic bodies (El Andaloussi et al., 2013b; Shao et al., 2018; Gurunathan et al., 2019). Here, we focus on the exosomes, which were first identified by Johnstone (Johnstone et al., 1987). Exosomes are cell secreted vesicles that can be isolated from various humor, such as blood, plasma, urine, tears, and lymph (Ibrahim and Marban, 2016), and they range in size from 30 to 150 nm.

A new cell-cell communication system has recently been identified, that lie with exosomes’ ability to target recipient cells and to transfer exosomal proteins, lipids, and nucleic acids to these cells (Milane et al., 2015; Zhang et al., 2015; Poe and Knowlton, 2018; Gurunathan et al., 2019). Exosomes have been shown to be closely related to tumorigenesis (Rodrigues et al., 2019), and exosomes have become a new type of clinical targets, for example, circulating exosomes containing glypican-1 have been used for the early judgment of early pancreatic cancer (Melo et al., 2015). Moreover, recent studies that exosomes are a potential system for targeted drug delivery (Kibria et al., 2018; Arrighetti et al., 2019; Sun et al., 2019).

The visual and auditory systems are the two major sensory systems. Many signaling pathways, such as Wnt, FGF pathway, are involved in the development of these two sensory systems (Heavner and Pevny, 2012; Żak et al., 2015). There are many factors that can lead to vision and hearing loss, and aging is a common cause of both. Hereditary factors are also important causes for hearing and vision loss. The Leber’s hereditary optic neuropathy is an inherited disease with mutations mitochondrial DNA that affects both visual and auditory functions (Rance et al., 2012; Bianco et al., 2017; Karanjia et al., 2017). Recent studies showed that exosomes can be secreted by visual and auditory cells (Han et al., 2017; Klingeborn et al., 2018; Breglio et al., 2020). Based on exosomes studies in other fields, exosomes might have similar uses in studying and treating disorders of the visual and auditory systems.

This article reviews the latest research in exosomes, described from exosome biogenesis, detection, and enrichment methods to their functions in visual and auditory systems.

## Biogenesis of Exosomes

**FIGURE 1:**
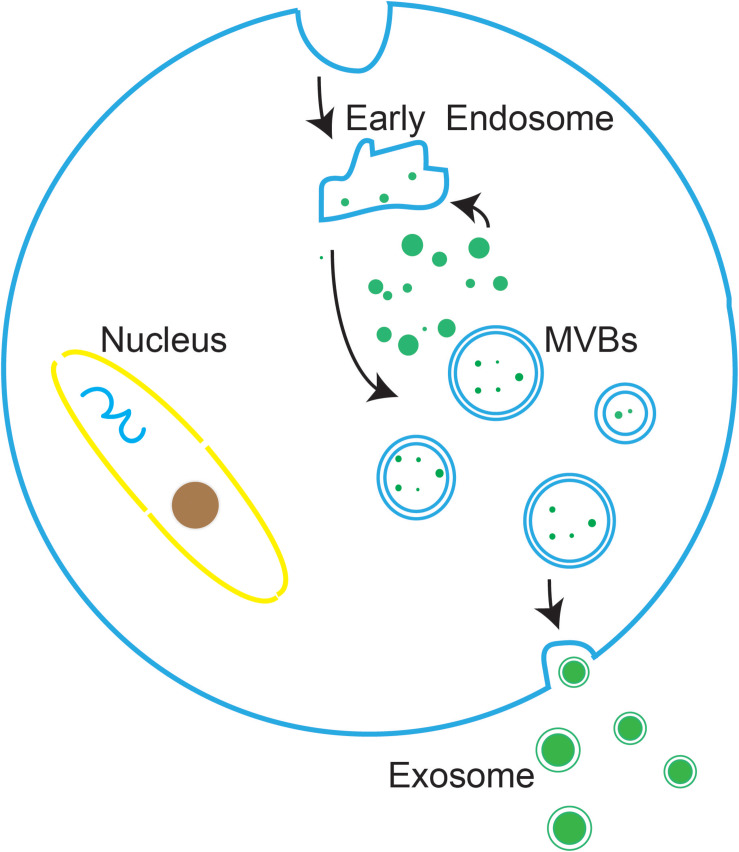
The biogenesis of exosomes. the plasma membrane invaginated to produced endocytic vesicles, which are further transformed into MVBs. MVBs then move and touch to the plasma membrane to secrete vesicles into the extracellular environment, at which point the vesicles are defined as exosomes.

Exosomes are vesicle-like bodies with a size of 30–150 nm that are produced by a variety of cells, and they have cup-shaped structures when observed under an electron microscope (Shao et al., 2012). Exosomes are originated from a number of type cells by the endosomal membrane pathway. First, the plasma membrane invaginated to produced endocytic vesicles, and multiple endocytic bodies then gather together to form early endosomes. Next, the invagination of early endosomes further transformed into multivesicular bodies (MVBs). Finally, MVBs move and touch to plasma membrane to secrete vesicles into the extracellular space, and these vesicles are defined as exosomes (Figure 1; Shao et al., 2018).

Exosome formation involves content sorting and release, and the precise regulation of this process requires the coordinated functions of many different proteins. Subunits of endosomal sorting complex required for transport (ESCRT) are necessary for exosome biogenesis, including ESCRT complexs from 0 to III (Farooqi et al., 2018). There are two mechanisms for exosome secretion, ESCRT-independent and ESCRT-dependent.

In the ESCRT-dependent mechanism, the ESCRT-0 complex recruit proteins by ubiquitin or clathrin. ESCRT-I and ESCRT-III control the budding process, the formation of ILVs, and the subsequent formation of exosomes. The ESCRT-independent mechanism involves syntenin, ALIX and ESCRT-III. Syntenin recruits proteins to form cargo cluster, and then ALIX, and ESCRT-III regulate the budding process, the formation of ILVs, and the subsequent formation of exosomes. The proteins recruitment and cargo clustering can be achieved by either the ESCRT-dependent or the ESCRT-independent mechanisms (van Niel et al., 2018).

## Compositions of Exosomes

Exosomes contain a variety of bioactive substances, mainly proteins, nucleotides, and lipids, and they are encapsulated by a phospholipid bilayer membrane that their internal bioactive substances avoid proteases degradation in the extracellular fluid (Tao et al., 2017). The phospholipid bilayer of exosomal membrane is composed of glycerophospholipids, sphingolipids, cholesterol, diglycerides, phospholipids and polyglycerophospholipids (Subra et al., 2010). Therefore, these exosomal proteins and nucleotides have potential use as reference index for disease judgment.

**FIGURE 2:**
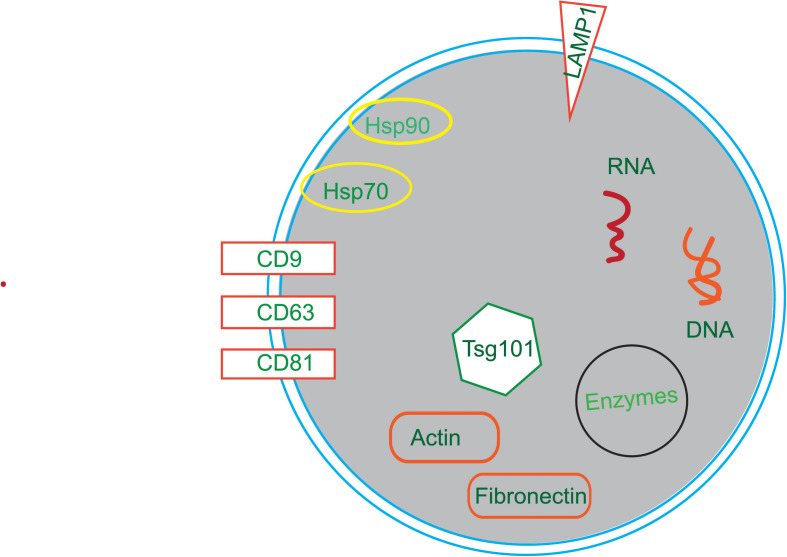
The compositions of exosomes. Exosomes are composed of in a variety of bioactive substances, mainly proteins, nucleotides and lipids. Proteins include tetraspanins (e.g., CD9, CD63, and CD81), heat shock proteins (e.g., Hsp70, Hsp90), cytoskeletal proteins (e.g., Actin, Fibronectin), viral proteins (e.g., Tsg101) and enzymes. Nucleotides include RNA and DNA.

Membrane transport and fusion proteins are the protein with the largest proportion on the surface of exosomes (Ibrahim and Marban, 2016; Farooqi et al., 2018), and these include tetraspanins (e.g., CD9, CD63, and CD81), heat shock proteins (e.g., Hsp70, Hsp90), cytoskeletal proteins (e.g., actin, fibronectin), and viral proteins (e.g., LAMP1, Tsg101). Other proteins also present in exosomes including signal transduction proteins, vesicle biogenesis proteins, and enzymes. Moreover, there are a variety of RNAs in exosomes, such as mRNAs, miRNAs, ncRNAs, and LncRNAs (Sexton et al., 2019; Figure 2).

## Isolation and Purification of Exosomes

The isolation and purification of exosomes shows significant promise for biomedical research (Witwer et al., 2013; Gardiner et al., 2016), but there are still difficulties to be solve in using exosomes in research and clinical applications. The main challenge is that the size and physicochemical properties of exosomes overlap with those of lipoprotein and protein complexes. Based on the size and biological characteristics of exosomes, many enrichment methods have been used to purify them, including differential ultracentrifugation, gradient ultracentrifugation, size-exclusion chromatography (Fonseca et al.), immunoaffinity enrichment, and co-precipitation. More speed and efficient methods are being exploited to purified exosomes.

### Ultracentrifugation

In cell culture medium, blood, urine, and breast milk, exosomes are often mixed with other impurities that have similar physical and chemical properties, including lipoproteins, cell fragments, etc. According to their specific size and density distribution range, exosomes can be effectively separated from these particles by ultracentrifugation and density gradient centrifugation. Ultracentrifugation is considered the most common traditional strategy for exosome enrichment (Gardiner et al., 2016). Combinations of different speeds and times are used in ultracentrifugation to remove other impurities in the sample step-by-step, and finally the separation and enrichment of exosomes are achieved. First, whole cells and large apoptotic bodies are depleted for 15–30 min by low-speed centrifugation (600–2,000 × *g*) at 4°C (Crewe et al., 2018; Langevin et al., 2019). Next, the supernatant centrifuged for 45–60 min by 12,000 × *g* centrifugation at 4°C to remove MVBs (Langevin et al., 2019). Lastly, the supernatant is moved to a new ultracentrifuge tube and centrifuged for 1–2 h by 100,000 × g-120,000 × *g* centrifugation at 4°C (Witwer et al., 2013; Langevin et al., 2019). The supernatant is deleted and the particles are resuspended in 100 μL phosphate-buffered saline. Exosomes cannot be separated completely by particle size using this protocol, because sedimentation is based on the density and other non-exosome vesicles can also be enriched (Zhang M. D. et al., 2018). EVs have a unique lipid membrane structure that encapsulates a certain amount of nucleic acids and proteins, resulting in a density range of 1.13–1.19 mg/ml (Thery et al., 2006). Density-gradient centrifugation involves centrifuging through an iodixanol or sucrose density gradient and different particles settle at different points in the gradient based on their different densities (Shao et al., 2018). Compared to differential centrifugation, the density-gradient centrifugation approach results in purer exosomes, but it requires a longer time to reach equilibrium and thus leads to greater damage to the instrument.

### Size-Exclusion Chromatography (SEC)

SEC is a chromatography technique that differentiate molecules in a solution depend on their size and molecular weight (Boing et al., 2014; Burgess, 2018). The purification column is made up of spherical beads with a specific aperture pores, and examples of commonly used materials are Sephadex, Sepharose, and Sephacryl. When the sample flows into column, large molecules block out the pores, while small molecules can diffuse into the pores. Therefore, larger molecules pass through the column faster than small molecules, and exosomes are separated due to their size. Purified exosomes can be isolated from complex biological media such as milk, urine, and plasma using SEC (Lozano-Ramos et al., 2015; Blans et al., 2017; Kreimer and Ivanov, 2017; Shao et al., 2018).

### Immunoaffinity Enrichment

Immunoaffinity enrichment is based on antibodies to specific exosome marker proteins. Proteins such as CD9, CD63, and CD81 are located on the exosome surface, and tumor-associated markers (HER2, EpCAM) are also present on tumor-associated exosomes (Taylor and Gercel-Taylor, 2008; Woo et al., 2016; Barok et al., 2018). Antibodies against these proteins linked with beads or other substrates by covalent or high-affinity interactions, and these antibodies bind to exosomes using low-speed centrifugation or magnetic techniques (Witwer et al., 2013). Taylor and Gercel-Taylor have successfully isolated circulating exosomes secreted from tumors using EpCAM magnetic beads (Taylor and Gercel-Taylor, 2008). This method has the potential for high specificity and efficiency (Tauro et al., 2012) and is usually performed using commercially available kits.

### Co-precipitation

Recently, polymer co-precipitation strategies have been exploited to enrich exosomes. The common methods are protamine precipitation, acetate precipitation, protein organic solvent precipitation, and hydrophilic polymers precipitation (Brownlee et al., 2014; Deregibus et al., 2016; Gallart-Palau et al., 2016). These reagents precipitate EVs by reducing the hydration and thus the solubility of EVs (Shao et al., 2018). Therefore, it is possible to isolate exosomes using low centrifugal forces. Based on this method, many exosome extraction kits have been developed, for instance Total Exosome Isolation (Invitrogen, United States).

### New Enrichment Methods

In the past few years, many exosome enrichment methods have been developed, here we summarize some of the recent progress.

Microfluidic filtering is a novel technology that extracts exosomes from a small amount of liquid (10^–9^ to 10^–18^ liters) through channels ranging from tens to hundreds of micrometers (Whitesides, 2006). With recent developments in nanomaterials, some emerging nanomaterials have been used for the capture of exosomes. For example, Lim et al. (2019) produced nanowires with CD63, CD9, and CD81 antibodies attached to their surfaces for capturing exosomes.

**FIGURE 3:**
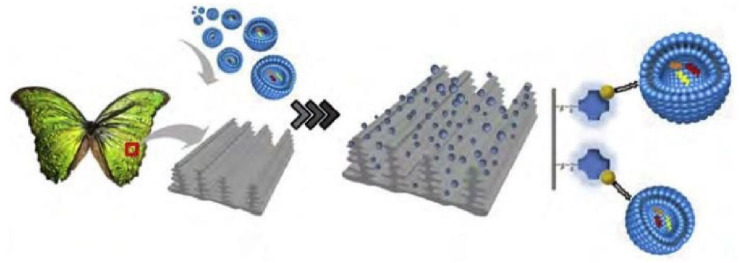
Scheme of isolation and enrichment of EVs by *M. Menelaus* wings. *M. Menelaus* wings were modified by lipid nanoprobes. When samples through *M. Menelaus* wings, the lipid nanoprobes can insert into the EV membranes to capture EVs.

Recently, Chai et al. developed a noval microvortex chips method using *Morpho Menelaus (M. Menelaus)* butterfly wings modified by lipid nanoprobe, which, when integrated into microfluidic chips, greatly improved the efficiency of EV enrichment by over 70%. *M. Menelaus* wings have an original three-dimensional (3D) microgroove structure linked with many intersection points, and these microgrooves are distributed on wing surface parallelly. Due to this structure of *M. Menelaus* wings and the lipid bilayer structure of EVs, the lipid nanoprobe modified *M. Menelaus* wings can be applied to isolate and purified EVs (Figure 3) (Han et al., 2020). These results demonstrated that the efficiency is greatly improved by using new microvortex chips. Based on this method, enrichment exosomes by *M. Menelaus* wings is possible. There are many exosomal marker proteins, which are present on the surface of exosome (e.g., CD9, CD63, and CD81). Based on M. *Menelaus* wings structure, antibodies of these marker proteins can be modified on M. *Menelaus* wings. When samples enter M. *Menelaus* wings, antibodies of these marker proteins will bind to marker proteins on exosome surface and thus capture exosomes effectively.

## Characterization and Detection of Exosomes

Exosomes not only have extremely small particle size, but also have a high heterogeneity and diversity among individual exosomes due to different cell sources, cell states, and secretory pathways (Tkach and Thery, 2016). Thus it is a challenge to accurately detect and characterize exosomes. Here we review some methods for the characterization and detection of exosomes.

### Scanning Electron Microscopy (SEM)

SEM is a common method to observe the morphology of exosomes (van der Pol et al., 2010; Sokolova et al., 2011). The surface of the exosome sample is scanned by an electron beam to produce the images. Information about the three-dimensional surface morphology and the elemental composition of the exosomes is obtained through various signals that result from the interaction between the electron beam and atoms in the sample (Shao et al., 2018). Under SEM, most exosomes show a cup-shaped morphology (Thery et al., 2006).

### Transmission Electron Microscopy (TEM)

TEM is another method commonly used for exosome characterization (Thery et al., 2006). Compared to SEM, TEM has superior resolution and can resolve objects less than 1nm in size. Using short wavelength electrons, TEM can also detect the cup-shaped morphology of exosomes. Exosome samples need to be stained with special chemical reagents such as osmium tetroxide and phosphotungstic acid in order to be observed by TEM (Shao et al., 2012).

### Nanoparticle Tracking Analysis (NTA)

NTA was first applied for exosome detection and characterization in 2011 (Sokolova et al., 2011). NTA is a technique using optical particle to track nanoparticles for measuring concentration and size distribution of the nanoparticles (Dragovic et al., 2011; Sokolova et al., 2011; Gardiner et al., 2013). The principle of NTA is that the random Brownian motion of nanoparticles in liquids can be recorded with a high-speed camera (Bachurski et al., 2019), and this information can be used in the Stokes-Einstein equation to determine the size and concentration of the tracked particles (Dragovic et al., 2011; Kestens et al., 2017). Compared with other methods, NTA is now apply for exosome analysis at the single particle level in many studies.

### Dynamic Light Scattering (DLS)

DLS is also lied with the Brownian motion of the particles, and this method can detect particle sizes ranging from 5 to 10 nm to 6 μm through fluctuations in the scattered light intensity (Erdbrugger and Lannigan, 2016). When particles do Brownian motion, the scattered light waves emitted by all particles interferes with each other, and their intensities change over time (Shao et al., 2018). The hydrodynamic radius of the particles is calculated by using the Siegert relationship to convert the intensity autocorrelation function into the scattering electric field correlation function (Sitar et al., 2015).

### Tunable Resistive Pulse Sensing (TRPS)

TRPS is an approach to quickly characterizing the particle size distribution and concentration of nanoparticles at the single particle level (Coumans et al., 2014; Vogel et al., 2016). The TRPS device has a membrane with a pore, and a current through the pore is produced by applying a voltage to the membrane (Blundell et al., 2015). The exosome sample is placed on one side of the membrane and only one particle is allowed to pass through the pore at a time under the drive force of the pressure and voltage difference (Koritzinsky et al., 2017). The particle concentration and size are determined by particle passing through the pore frequency and the drop in current, respectively.

### Western Blotting and Enzyme-Linked Immunosorbent Assay (ELISA)

Proteins are one of the main components of exosomes, and the quantification and identification of exosomal proteins is essential not only for explaining exosome biogenesis and transport, but also for identifying disease markers (Shao et al., 2018). Western blotting and ELISA are conventional protein analysis techniques. Western blotting, also called immunoblotting, is the most common protein assay approach and is widely used in molecular biology research. This process relies on antibody-protein interactions and generally apply to detect the existence of target proteins in exosomes.

ELISA is another available approach for protein quantification. ELISA also relies on antibody-protein interactions, but it uses a specific antibody attached to an enzyme. The antigen and antibody are directly proportional to the antibody, and the absorbance of the enzyme is measured to quantify the protein (Engvall and Perlmann, 1971; Aydin, 2015; Knight et al., 2018). Compared to western blotting, ELISA can be faster and scaled up for high throughput measurements.

### Mass Spectrometry (MS)

MS has high specificity and sensitivity, can not only recognize and characterize the molecular components of vesicles, but can also be used for high-throughput peptide profiling (Kreimer et al., 2015; Pocsfalvi et al., 2016). Sample-preparation for proteomics analysis normally follows three steps: (1) SDS-PAGE separation (Lin et al., 2004; Pisitkun et al., 2004), (2) isoelectric focusing-based fractionation (Choi et al., 2012), and (3) two-dimensional liquid chromatography (Gonzalez-Begne et al., 2009). Over the past decade, the integration of qualitative MS applications and quantitative protein analysis has been greatly improved in terms of sensitivity, resolution, and speed. There are two main technical methodologies for MS quantitation: label-based and label-free quantitation (Choi et al., 2015). Label-free quantitation is more widely applied than label-based.

### New Detection Methods

As the development of exosome detection technologies have developed in recent years, more and more studies have focused on the detection of single vesicles. Total internal reflection fluorescence microscopy, based on aptamer fluorescent DNA nanodevice on target exosome surfaces, is an ultra-sensitive method that has been developed to directly quantify and visualize tumor exosomes in plasma samples at the single-vesicle level (He et al., 2019). Localized surface plasmon resonance imaging platform detects single exosome by using a nano-microarray with gold sensing element atop quartz nanopillars binding anti-CD63 (Raghu et al., 2018). Single particle interferometric reflectance imaging sensor, which is based on interference reflectance, and requires LED lights and a COMS camera, is used to count exosomes (Daaboul et al., 2016).

## Applications of Exosomes

Exosomes mediate a number of biological processes, including physiological and pathological processes. Exosomes contain various important biomolecules (e.g., proteins and RNAs) that regulate intercellular communications, and clinical applications of exosomes have also achieved breakthroughs (Thery, 2015; Meldolesi, 2018; Huang and Deng, 2019). Currently, most of the exosome research is focused on the tumors, and it has been identified that exosomes encourage cell polarity and control cell motility by altering extracellular matrix (ECM) components (Sung et al., 2015). Exosomes can also stimulate angiogenic activities (Zhang et al., 2016), and tumor-derived exosomes can activate the immune response (Greening et al., 2015). In contrast to research in tumors, few studies have focused on exosomes in diseases of the eye and the inner ear. Based on the roles of tumor-derived exosomes, eye cells-derived exosomes might play similar roles. Consequently, we summary the applications and exosomes’ functions reported in the visual and auditory systems.

### Exosomes in the Visual System

The eye is a vital sensory organ, and eye diseases can have significant negative impacts on daily life. Age-related macular degeneration, cataract, diabetic retinopathy, and glaucoma are the four most widespread eye diseases (Saldanha et al., 2017). Aging is the main cause of eye disease, and age-related visual impairment involves corneal endothelial cells’ death, a decline in the number and sensitivity of neurons in the retina, and changes in the tissue structure of eye (Lei et al., 2011; Gambato et al., 2015; Nadal-Nicolás et al., 2018; Wang et al., 2018).

Exosomes have been identified promote cells and the ECM communication and to participate in ECM assembly and adhesion (Hoshino et al., 2013; Mead et al., 2013; Wang et al., 2017). Glaucoma is a common eye disease, and its pathogenesis involves the ECM of the trabecular meshwork (TM), oxidative stress, the TGFβ signaling pathway, and apoptosis (Suri et al., 2018). Human TM explants exposure in dxamethasone induces fibronectin production, and reduces the interaction of exosomes for the fibronectin surface, and this might explain the abnormal amassing of ECM substances in steroid-induced glaucoma patients (Dismuke et al., 2016). Studies have shown that alters in the ECM in the lamina cribrosa and TM are behind the irreparable vision loss in glaucoma (Klingeborn et al., 2017), and illumination the exosome-related defects in the ECM in both tissues will likely lead to a better explanation of the pathogenesis of glaucoma.

Exosomes have been shown effect immune regulation in many diseases, including tumors (Seo et al., 2018), inflammatory diseases (Cypryk et al., 2018), autoimmune diseases (Anel et al., 2019), and neurodegenerative disorders (Tofaris, 2017). However, there have been few studies showing the roles of exosomes in immune regulation in eye diseases. Corneal transplantation is an effective treatment for blindness, but immune rejection is still a significant problem, and studies have indicated that exosomes are involved in recovery process after corneal transplantation (Coulson-Thomas et al., 2013; Harrell et al., 2018). Short collagen-like peptides (CLPs) such as CLP-PGE promote the regeneration of stable corneal tissues and nerves by stimulating the production of exosomes by corneal epithelial cells (Jangamreddy et al., 2018). In addition, mouse-derived exosomes have been identified that as a medium, involved in signal transmission between cells during corneal wound healing (Han et al., 2017).

Mesenchymal stem cell (MSC) derived exosomes show therapeutic potential in various diseases. MSC-derived exosomes can relieve the symptoms of type two diabetes mellitus (Sun et al., 2018), and hypoxia-conditioned bone marrow MSC-derived exosomes can promote heart repair after ischemic injury by reducing myocardial apoptosis (Zhu et al., 2018). Similarly, MSC-derived exosomes have been identified make significant contributions in the visual system. The degree of *in vitro* wound healing by corneal MSCs (cMSCs) of human corneal epithelial cells is higher than controls, and corneas treated *in vivo* with cMSC-derived exosomes also have a higher degree of wound healing (Samaeekia et al., 2018). In the rat retinal detachment model, MSC-derived exosomes can inhibit apoptosis of photoreceptor cells and maintain normal retinal structure (Ma et al., 2020). MSC-derived exosomes inhibit the migration of inflammatory cells and decline the infiltration of white blood cells to ease the progress of experimental autoimmune uveitis (Bai et al., 2017). MSC-derived exosomes also improve visual function by downregulating the vascular endothelial growth factor-A produced (He et al., 2018).

Exosomes not only participate in pathological and physiological processes, but also have therapeutic and diagnostic potential. Adeno-associated virus (AAV), a small, non-enveloped virus (Samulski and Muzyczka, 2014), is often used as a gene therapy carrier with its high efficiency and safety (Wassmer et al., 2017). Exosomes (exo) can be combined with AVV to produce exo-AAVs that have higher gene transfer efficiency compared to AVVs alone (Hudry et al., 2016). Wassmer et al. packaged the green fluorescent protein gene into AAV2 and exo-AAV2 vectors and injected them into mouse eyes through intravitreal (IVT). The data indicated that the exo-AAV2 vector was superior compare to AAV in terms of delivery and retinal transduction and that exo-AAV2 could enhance gene transduction in the ganglion cell layer and in the deep retina (Wassmer et al., 2017). Owing to exosomes can pass across the blood-brain barrier, and the endothelial barrier (El Andaloussi et al., 2013a; Hudry et al., 2016), they might provide a chance to treat of eye diseases.

Exosomal RNA is significantly increased in both myopic and normal aqueous humor (AH) samples, and there are several specific miRNAs that might be useful biomarkers of myopia (Chen et al., 2019). Exosomes are widely found in all kinds of fluids, including tears, so it might be possible to find exosomal markers of in eye diseases from tears. Some studies have identified the appearance of exosomal marker proteins in tears, such as CD9, CD63 (Aass et al., 2015; Matheis et al., 2015; Grigor’eva et al., 2016).

### Exosomes in Hearing System

Deafness is a common sensory disease and sensing hair cells (HCs) in the inner ear are sensors for recognizing sound. In mammals, HC loss due to noise, aging, and ototoxic drugs is irreparable and is thus the major cause of permanent hearing loss (Brigande and Heller, 2009; Furness, 2015). Recent studies showed that exosomes are present in the inner ear, and the release of inner ear exosomes is decreased and the protein profile of exosomes is significantly changed after treatment with ototoxic drugs such as neomycin and cisplatin (Wong et al., 2018). Exo-AAV has been successfully used as a non-toxic and noval gene vector for gene therapy of retinal diseases in mouse models, and György et al. have demonstrated that exo-AAV is also an effective gene vector for inner ear HCs (György et al., 2017). The addition of the exo-AAV vector to the *in vitro* culture medium showed that about 95% of the vector was transduced into inner and outer HCs (IHCs and OHCs). The vector was also injected into the mouse cochlea *in vivo* through round window (RW), and the transduction efficiency in IHCs and OHCs was 88 and 25%, respectively. LHFPL5, also called Tmhs, is an essential component of the mechanical transduction mechanism of OHCs and IHCs, and its deletion can cause hearing loss (Xiong et al., 2012). Exo-AAV- *HA-Lhfpl5* injection through RW could partically rescue hearing in *Lhfpl5-/-* mice (György et al., 2017), thus exo-AAV vectors might provide a new method in gene therapy for treating clinical deafness.

Exosomes can mediate intercellular communication between different cells by delivery of exosomal proteins, nucleic acid and lipids to recipient cells (Colombo et al., 2014). Heat-shock 70-kDa protein (HSP70) is widely found in exosomes (Fonseca et al., 2016), and previous study by Lisa L Cunningham et al. showed that supporting cells (SCs) require HSP70 to protect HCs (May et al., 2013). Recently, they found that SCs derived exosomes are significantly increased by heat shock, and these exosomes can improve promote HCs survival under neomycin exposure. Furthermore, they found that HSP70 in SCs-derived exosomes interact with TLR4 receptors on the cell membrane of HCs, which can protect HCs from damage (Breglio et al., 2020).

Exosomes are likely associated with the pathological processes of inner ear diseases, for instance sensorineural hearing loss and genetic deafness. At present, although there are only a few reports on exosomes in the auditory system, we believe that exosomes will make significant contributions in future clinical treatment of inner ear diseases and prediction of deafness.

## Conclusion and Perspectives

Exosomes contain abundant proteins and nucleic acids, and they are considered to be a medium for transmitting information between cells. Exosomes have significant functions not only in the metastasis and growth of tumor cells, but also in normal physiological processes. The phospholipid bilayer structure and rich proteins and nucleic acids of exosomes also make them useful as biomarkers for medical diagnose. Due to their heterogeneity and not restricted by the blood-brain barrier, exosomes can design as targeted drug-delivery vehicles, and will likely be important components of novel therapeutic strategies.

Many new methods for analyzing exosomes have been developed in recent years, but the isolation and analysis of exosomes remains a challenge because the molecular interactions and the precise functions of exosomes are still difficult to analyze. New technologies for single exosome analysis are required to reveal the unique molecular functions and diversity of exosomes. Exosomes exist not only in various body fluids, but also in tissues, and the requirement for extraction specified steps are different in different samples. Therefore, further studying the functions of exosomes in disease processes, it is vital to establish standard protocols based on different exosomal sample types to be extracted to avoid influence from human normal physiological factors.

**FIGURE 4:**
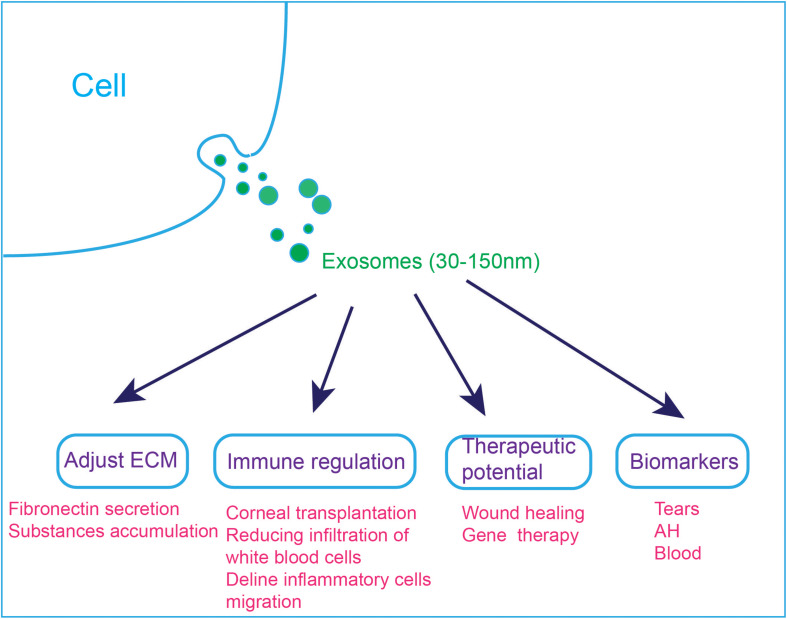
Applications of exosomes in the visual and auditory systems. (1) Exosomes are involved in cell ECM changes. Dexamethasone treatment decreases the affinity of exosomes to fibronectin, which can explain the abnormal accumulation of ECM substances in steroid-induced glaucoma patients. (2) Exosomes are involved in immune regulation, and they can reduce immune rejection after corneal transplantation. MSC-derived exosomes ameliorate autoimmune uveoretinitis by inhibit the migration of inflammatory cells and decline the infiltration of white blood cells into the eye. (3) Exosomes can accelerate wound healing and exo-AAVs are considered to be a novel gene therapy tool. (4) Exosomes in tears, AH and blood have the potential to be used as diagnostic markers.

Research on exosomes in the visual and auditory systems is still a relatively young field, especially the field of hearing, and the related research is summarized in Figure 4. To further study exosomes in the field of vision and hearing, it is necessary to develop animal models for exosom research, such as mouse, zebrafish or *Drosophila* models. With the help of animal models, we can more clearly explain the function of exosomes. It was reported that exosome reporter mice has been used to show exosomes involved in central nervous system communication (Men et al., 2019).

The discovery of more and more exosomal disease markers and the chance as a novel drug delivery vehicle are expected to apply in disease judgment, and thus improve patients’ outcomes.

## Author Contributions

PJ and SZ wrote the manuscript. CC, SG, and MT collated the resource. LL, GY, and RC wrote and reviewed the manuscript.

## Conflict of Interest

The authors declare that the research was conducted in the absence of any commercial or financial relationships that could be construed as a potential conflict of interest.
